# Clinician-rated mental health in outpatient child and adolescent mental health services: associations with parent, teacher and adolescent ratings

**DOI:** 10.1186/1753-2000-4-29

**Published:** 2010-11-25

**Authors:** Ketil Hanssen-Bauer, Øyvind Langsrud, Siv Kvernmo, Sonja Heyerdahl

**Affiliations:** 1 Centre for Child and Adolescent Mental Health, Eastern and Southern Norway, P.O. Box 4623 Nydalen, NO-0405 Oslo, Norway; 2 Department of Research and Development, Division of Mental Health Services, Akershus University Hospital, Lørenskog, Norway; 3 The Regional Centre for Child and Adolescent Mental Health - North, Institute of Clinical Medicine, Faculty of Health Sciences, University of Tromsø, Norway; 4 Department of Child and Adolescent Mental Health, Division of Child and Adolescent Health, University Hospital of North Norway, Norway

## Abstract

**Background:**

Clinician-rated measures are used extensively in child and adolescent mental health services (CAMHS). The Health of the Nation Outcome Scales for Children and Adolescents (HoNOSCA) is a short clinician-rated measure developed for ordinary clinical practice, with increasing use internationally. Several studies have investigated its psychometric properties, but there are few data on its correspondence with other methods, rated by other informants. We compared the HoNOSCA with the well-established Achenbach System of Empirically Based Assessment (ASEBA) questionnaires: the Child Behavior Checklist (CBCL), the Teacher's Report Form (TRF), and the Youth Self-Report (YSR).

**Methods:**

Data on 153 patients aged 6-17 years at seven outpatient CAMHS clinics in Norway were analysed. Clinicians completed the HoNOSCA, whereas parents, teachers, and adolescents filled in the ASEBA forms. HoNOSCA *total score *and nine of its scales were compared with similar ASEBA scales. With a multiple regression model, we investigated how the ASEBA ratings predicted the clinician-rated HoNOSCA and whether the different informants' scores made any unique contribution to the prediction of the HoNOSCA scales.

**Results:**

We found moderate correlations between the total problems rated by the clinicians (HoNOSCA) and by the other informants (ASEBA) and good correspondence between eight of the nine HoNOSCA scales and the similar ASEBA scales. The exception was HoNOSCA scale 8 *psychosomatic symptoms *compared with the ASEBA s*omatic problems *scale. In the regression analyses, the CBCL and TRF *total problems *scores together explained 27% of the variance in the HoNOSCA *total scores *(23% for the age group 11-17 years, also including the YSR). The CBCL provided unique information for the prediction of the HoNOSCA *total score*, HoNOSCA scale 1 *aggressive behaviour*, HoNOSCA scale 2 *overactivity or attention problems*, HoNOSCA scale 9 *emotional symptoms*, and HoNOSCA scale 10 *peer problems; *the TRF for all these except HoNOSCA scale 9 *emotional symptoms; *and the YSR for HoNOSCA scale 9 *emotional symptoms *only.

**Conclusion:**

This study supports the concurrent validity of the HoNOSCA. It also demonstrates that parents, teachers and adolescents all contribute unique information in relation to the clinician-rated HoNOSCA, indicating that the HoNOSCA ratings reflect unique perspectives from multiple informants.

## Background

Many child and adolescent mental health services (CAMHS) have established routine outcome measurement systems at the service level [1]. These often include broad measures of mental health symptoms, problems, and functioning rated by several informants, such as parents, teachers, and young people [2-4], or by clinicians [5-7]. These assessments require empirical evidence of their acceptable reliability, validity, feasibility, and sensitivity to change when used in routine outcome evaluations [8]. In the absence of gold standard criteria, we can assess the validity of a measure by investigating its correspondence with comparable measures [9].

The Health of the Nation Outcome Scales for Children and Adolescents (HoNOSCA) is an outcome measure rated by clinicians. It is a brief, quickly completed instrument that measures broad aspects of mental health problems and functional impairment. The HoNOSCA was established as a mandatory routine outcome measure of CAMHS in Australia [10], New Zealand [11], and Denmark [12], and has been widely used in the United Kingdom [13]. Several studies have concluded that it is a valid, reliable, and change-sensitive measure [7,14-19], and several studies have specifically examined the concurrent validity of HoNOSCA [20]. The correlations between the HoNOSCA *total score *and other clinician-rated measures, such as the Children's Global Assessment Scale (*r *= -0.35 [21] and *r *= -0.64 [18]), the Global Assessment of Psychosocial Disability (*r *= 0.46) [12], and the Paddington Complexity Scale (*r *= 0.46 [22] and *r *= 0.62 [18]) have been medium to large. Clinicians make important contributions to mental health assessments, and they require information about their patients' behaviour and functioning from the patients themselves or from people who know them. There are several potential sources of systematic error in clinicians' judgments, which may include personal interests if their assessments are used for outcome evaluations. Because clinicians' judgments could be biased, we wanted to study the associations between clinicians' HoNOSCA ratings and the ratings by parents, teachers, and adolescent patients themselves.

Medium correlations have been reported between the HoNOSCA *total score *and the Strengths and Difficulties Questionnaire (SDQ) *total difficulties score *[23] by parents (*r *= 0.38 [24] and 0.40 [18]), by teachers (*r *= 0.46 [24]), and by young people (*r *= 0.36 [24]). Medium correlations were also found when the HoNOSCA *total score *was compared with the Achenbach System of Empirically Based Assessment (ASEBA) forms: the Child Behavior Checklist (CBCL; parent report) *total problems *(*r *= 0.39) and the Teacher's Report Form (TRF) *total problems *(*r *= 0.35) [25]. However, further aspects of the concurrent validity of the HoNOSCA scales in routine clinical use must be investigated, to determine particularly whether they correlate, as expected, with similar scales of measures-rated by parents, teachers and adolescent patients.

The ASEBA is an integrated system of multi-informant assessment that is widely and routinely used in CAMHS. The 2001 versions of the CBCL and TRF are designed for subjects aged 6-18 years, and the Youth Self-Report (YSR) is designed for young people aged 11-18 years [26]. The three ASEBA forms have similar questions and scales, which differ from the HoNOSCA scales. In the ASEBA forms, the respondents assess many, very specific behaviours, whereas in the HoNOSCA, the clinician rates the clinical severity of the symptoms and problems on 13 scales. Although there are considerable differences between the instruments in both their format and content, there are substantial similarities in the themes that are addressed.

Modest levels of inter-informant agreement (small correlations) in paired comparisons of the ratings of behavioural problems by parents, young people, and teachers are robust findings, and it has been concluded that "each type of informant typically contributes a considerable amount of variance not accounted for by the others" [27]. As a consequence, multi-informant strategies are generally recommended as more valid than single-informant strategies for measuring mental health problems [4,28]. As far as we know, only one previous study has compared HoNOSCA and ASEBA in a clinical setting. This study was published by Brann as a dissertation (PhD) in 2006 [25].

In the study presented here, we first investigated correlations between presumed corresponding scales from the HoNOSCA and the multi-informant ASEBA (CBCL, TRF, and YSR). We chose the ASEBA because it is widely used to assess the mental health of children and adolescents, and because many of the ASEBA scales and syndromes address similar aspects of mental health to those addressed by the HoNOSCA scales. We expected higher correlations between scales that assessed similar phenomena than between scales that assessed less similar phenomena. Second, we used regression analyses to investigate how well the ratings by each ASEBA informant (CBCL, TRF, and YSR) predicted the clinician-rated HoNOSCA scores, and how well these ASEBA informants' scores together predicted the HoNOSCA scores. Specifically, we investigated which informants' scores provided the best prediction for the different HoNOSCA scales and whether the different informants' scores made any unique contribution to the prediction of the HoNOSCA scores.

## Method

### Procedures

Seven Norwegian outpatient CAMHS clinics participated in the study, which was part of a larger project to evaluate the HoNOSCA in routine use. The clinics had started to use the HoNOSCA as routine measures. We wanted the clinics to follow their ordinary routine practice, but we asked them to collect ASEBA forms as part of our research protocol. Four clinics recruited patients from January 2003 to November 2004, one from January 2003 to April 2006, and two from January 2005 to April 2006. The transfer of data to the project was based on the informed consent of the parents and adolescents. Patients acutely referred or who had problems with the Norwegian language were not included in the study. Only patients in the age group 6-17 years for whom a valid HoNOSCA, CBCL, and TRF was completed were included for analysis in the present study. The clinical staff at the outpatient CAMHS clinics rated the HoNOSCA after the first few assessment sessions. The rating was based on the two-week period preceding outpatient care.

The ASEBA forms (CBCL, TRF, and YSR) from 2001 [26] were given to the parents and the young people 11 years or older at the first meeting. The parents gave the TRF to the patients' teachers. The forms were collected at one of the next meetings (or sent by post). The informants or the clinicians sometimes filled in the measures late, and only ASEBA forms completed within 60 days before or after the clinician had rated the HoNOSCA were accepted, with a maximum of 90 days between any ASEBA forms. We did not give instructions to the clinicians about their clinical use of the ASEBA, and we have no information about whether the clinicians used the ASEBA information when they scored the HoNOSCA. However, we do know whether the ASEBA profile reports were available from the Assessment Data Manager (ADM) software [29] at the time the clinician rated the HoNOSCA.

### Measures

#### HoNOSCA

The HoNOSCA was developed in the United Kingdom to measure mental health and outcomes in clinical settings [14,30]. The HoNOSCA focuses on clinically significant problems and symptoms, and consists of 15 scales, each rated from 0 (no problem) to 4 (severe to very severe problem). The HoNOSCA *total score *is the sum of the first 13 scales (Table 1) and indicates the severity of the mental health problems. Because scales 14 and 15 focus on lack of knowledge about the child's condition and lack of information about appropriate services, they were not used in this study. The clinics arranged standard training in the use of HoNOSCA for their clinicians before and during the data collection period. The clinicians at five of the seven clinics participated in a larger study of the inter-rater reliability of the HoNOSCA, involving 169 clinicians from 10 outpatient CAMHS. The results of that reliability study have been described in more detail elsewhere [16], but the inter-rater reliability was found to be substantial for the HoNOSCA *total score *with an intraclass correlation coefficient (ICC) of 0.81. The reliability of the HoNOSCA was lowest for scale 6 *somatic problems *(ICC = 0.47), scale 8 *psychosomatic problems *(ICC = 0.59), scale 5 *scholastic problems *(ICC = 0.60), and scale 12 *family problems *(ICC = 0.60). The reliability was highest for scale 1 *aggressive behaviour *(ICC = 0.82), scale 3 *self-injury *(ICC = 0.90), scale 13 *poor school attendance *(ICC = 0.91), and scale 4 *drug or alcohol misuse *(ICC = 0.96).

**Table 1:** HoNOSCA scales and similar ASEBA scales or items

The HoNOSCA scales		Similar ASEBA scales or items
1	Problems with disruptive, antisocial, or aggressive behaviour	Broad-band scale: Externalizing problems
2	Problems with overactivity, attention, or concentration	Syndrome scale: Attention problems
3	Non-accidental self-injury	Item 18: Deliberately harms self or attempts suicide Item 91: Talks about killing self
4	Problems with alcohol, substance/solvent misuse	Item 2: Drinks alcohol without parents' approval (CBCL, YSR, but not TRF) Item 105: Uses drugs for non-medical purposes
5	Problems with scholastic or language skills	-
6	Physical illness or disability problems	-
7	Problems associated with hallucinations, delusions, or abnormal perceptions	Item 9: Can't get mind off thoughts Item 34: Others out to get him/her Item 40: Hears thing Item 70: Sees thing Item 85: Strange ideas Item 89: Suspicious
8	Problems with non-organic somatic symptoms	Syndrome scale: Somatic complaints
9	Problems with emotional and related symptoms	Broad-band scale: Internalizing problems
10	Problems with peer relationships	Syndrome scale: Social problems
11	Problems with self-care and independence	-
12	Problems with family life and relationships	-
13	Poor school attendance	Item 98: Tardy to school or class (TRF, but not CBCL or YSR) Item 101: Truancy, skips school
HoNOSCA total score (sum scale 1-13)		Broad-band scale: Total problems

#### ASEBA

The 2001 version of the ASEBA forms [26] were used: CBCL for ages 6-18 years, YSR for ages 11-18 years, and TRF for ages 6-18 years. The questionnaires contain 120 items regarding behavioural and emotional problems, which are scored 0 (not true), 1 (somewhat or sometimes true), or 2 (very true or often true). The ratings are based on the past six months for the CBCL and YSR and for the past two months for the TRF. No form was accepted as valid if there were more than eight missing items. For the CBCL, YSR and TRF, we computed the eight syndrome scales (*anxious/depressed, withdrawn/depressed, somatic complaints, social problems, thought problems, attention problems, rule-breaking behaviour *and *aggressive behaviour*), and the broadband scales (*internalizing problems, externalizing problems*, and *total problems*), as described by Achenbach et al. [26].

### Similar symptoms and problems identified with the HoNOSCA and ASEBA

We compared the total scores for the two methods. We also compared the HoNOSCA scales with the ASEBA scales that we found to be similar in content (Table 1). The HoNOSCA scale 3 *self-injury*, scale 4 *drug or alcohol misuse*, scale 7 *abnormal thoughts or perceptions*, and scale 13 *poor school attendance *were not similar to any scales in the ASEBA. However, there were relevant items in the ASEBA, and we made a sum score for the relevant ASEBA items for the correlation analysis (Table 1). HoNOSCA scale 3 *self-injury *and HoNOSCA scale 4 *drug or alcohol misuse *were rated zero (no problem) for all children in the age group 6-10 years; HoNOSCA scale 7 *abnormal thoughts or perceptions *was rated zero for 92% in this youngest age group, and HoNOSCA scale 13 *poor school attendance *was rated zero for 90% of them. Therefore, we performed correlation analyses with these scales only in the oldest age group (11-17 years).

### Descriptions of the sample

The sample comprised 153 patients, all with a valid HoNOSCA, CBCL, and TRF. The mean age was 11.5 years (SD 3.0, range 6-17 years), which ranged between the clinics from 9.5 to 12.7 (F = 2.4, d.f. = 6, *P *= 0.031). The proportion of girls was 46%, and this did not differ between clinics (χ^2 ^= 7.1, d.f. = 6, *P *= 0.310). The girls had a mean age of 12.5 (SD 3.0) and the boys of 10.7 years (SD 2.7), which were significantly different (*t *= 3.9, d.f. = 151, *P *< 0.001). Seventy-five (82%) of the 92 patients in the age group 11-17 years had a valid YSR. These responding and non-responding young people did not differ in their *total problems *scores on the HoNOSCA (*t *= 0.64, d.f. = 90, *P *= 0.525), CBCL (*t *= 1.46, d.f. = 90, *P *= 0.147) or TRF (*t *= 1.13, d.f. = 90, *P *= 0.262). Forty-one (55%) of the 75 young people who responded were girls. One clinic provided data on 80 of the 153 patients in the sample, and the other six clinics had between four and 22 patients each, indicating a very low inclusion rate for some of the clinics. We did not have clear information on the response rates. The reasons for non-inclusion were: one or more measures missing, acute referral, language problems, lack of consent, early drop-out or discharge, or the clinician did not follow the protocol correctly. We had HoNOSCA scores for 288 patients. The sample comprised 153 of those patients for whom we had valid CBCL and TRF scores. The mean HoNOSCA *total score *for the 135 patients without a valid CBCL or TRF did not differ from that of the 153 patients included in the present sample (*t *= 0.11, df = 286, *P *= 0.911). These 153 patients were rated by 51 different clinicians, with a range of 1-28 patients per clinician and a range of 2-13 clinicians per clinic (mean 7.1, SD 3.3). Fifteen patients were rated after the clinicians had discussed their case with a colleague, and 102 patients were rated by a clinician with no discussion (missing data for 36 patients). One hundred and fifteen of the 153 patients (75%) were scored by a clinician with previous training in the use of the HoNOSCA (missing data for five patients). The clinicians included 22% psychologists, 14% medical doctors, 15% social workers, 37% educational therapists, and 12% with another bachelor degree.

We used the CBCL form completed by the biological mother if available (*n *= 134); if not, we used the form completed by the biological father (*n *= 11). We used the CBCL forms received from the foster mothers of six patients, who had no form from a biological parent. Two parents in the sample had filled in the form without giving further information about the relationship. If more than one teacher had completed the form, we selected the form from the teacher who had most contact with the pupil.

The mean time from when the CBCL was completed to when the HoNOSCA was rated (date of HoNOSCA rating minus date of CBCL rating) was 5.5 days (SD 24.4 days), the mean time from the TRF to the HoNOSCA was -1.8 days (SD 22.4 days), and the mean time from the YSR to the HoNOSCA was 0.3 days (SD 22.7 days). The mean time difference (ignoring the order) between the HoNOSCA and CBCL was 18.2 days (SD 17.1), that between the HoNOSCA and TRF was 16.8 days (SD 14.8), and that between the HoNOSCA and YSR was 15.7 days (SD 16.2).

Table 2 shows the descriptive statistics, with the sex and age group effects, for the HoNOSCA scales. The mean HoNOSCA *total score *was 12.0 (SD 4.6). Eighty-four per cent of the patients had a score of 3 or 4 (severe problems) on one or more scales: 28% had a score of 3 or 4 on one scale, 25% on two scales, 19% on three scales, 8% on four scales, 3% on five scales, 1% on 6 scales and none on 7 or more scales. The mean number of scales with a score of 3 or 4 was 1.9 (SD 1.4). Table 3 shows the descriptive statistics for the eight ASEBA syndrome scales and the broader *internalizing problems*, *externalizing problems*, and *total problems *for the CBCL, YSR, and TRF. Because many patients (44%, *n *= 67) had severe scores (3 or 4) on only one or no scales, we examined the ASEBA scores for this group. They had mean CBCL *total problems *39.6 (SD 22.2), mean TRF *total problems *37.2 (SD 27.9), mean YSR *total problems *41.2 (SD 28.2) and mean HoNOSCA *total score *8.6 (SD 2.9).

**Table 2:** HoNOSCA scales scores.

			**Effects**^**1**^		**Score distribution**^**2**^				
	
	Mean (SD)		Sex	Age	0	1	2	3	4
1. Aggressive behaviour	1.2	(1.1)	B > G*		33%	26%	24%	16%	1%
2. Overactivity or attention problems	1.9	(1.3)	B > G*		22%	12%	18%	46%	3%
3. Self-injury^3,4^	0.2	(0.7)	G > B**	O > Y**	88%	5%	4%	4%	0%
4. Drug or alcohol misuse^3^	0.1	(0.4)		O > Y**	90%	8%	2%	1%	0%
5. Scholastic problems	1.5	(1.3)	B > G*		32%	17%	23%	25%	3%
6. Somatic problems	0.5	(0.9)			71%	11%	14%	2%	1%
7. Abnormal thoughts or perceptions	0.3	(0.7)			86%	7%	5%	3%	0%
8. Psychosomatic symptoms	0.8	(1.0)			54%	24%	12%	9%	1%
9. Emotional symptoms	1.6	(1.1)	G > B**		22%	25%	22%	31%	0%
10. Peer problems	1.5	(1.1)			24%	29%	26%	18%	3%
11. Self-care problems	0.4	(0.8)		Y > O**	73%	14%	10%	3%	1%
12. Family problems	1.5	(1.1)			26%	22%	35%	14%	3%
13. Poor school attendance	0.5	(0.9)		O > Y*	77%	5%	14%	3%	1%
Total Score (sum scale 1-13)	12.0	(4.6)							

Mean (SD), effect of sex, age, and score distribution (n = 153) on the HoNOSCA scales.

*P < 0.05 and false discovery rate (FDR) < 0.06, **P < 0.01 and FDR < 0.03, G = Girls, B = Boys, Y = Younger (6-10 years), O = Older (11-17 years).

^1 ^Effects were analysed using the general linear model (GLM) in SPSS, and the significant effects are shown.

^2 ^Scores: 0 = no problem; 1 = minor problem requiring no action; 2 = mild problem but definitely present; 3 = moderately severe problem; 4 = severe to very severe problem.

^3 ^All participants younger than 11 years were rated 0.

^4 ^Interaction Sex × Age is significant for this scale with *P *< 0.01 and FDR < 0.06

**Table 3:** ASEBA scales scores.

	CBCL (*n *= 153)				TRF (*n *= 153)				YSR (*n *= 75)		
ASEBA	Mean (SD)		**Effects**^**1**^		Mean (SD)		**Effects**^**1**^		Mean (SD)		**Effects**^**1**^
Syndrome Scales:			Sex	Age			Sex	Age			Sex
Anxious/depressed	6.2	(4.9)			4.8	(4.4)			7.1	(6.0)	G > B***
Withdrawn/depressed	3.7	(3.2)		O > Y*	3.0	(3.1)		O > Y***	4.5	(3.6)	G > B***
Somatic complaints	3.4	(3.1)	G > B**		1.4	(2.2)			3.9	(3.9)	G > B**
Social problems	5.2	(3.7)			3.5	(3.3)	B > G*		4.4	(3.8)	
Thought problems	3.1	(2.9)			1.3	(1.9)	B > G*		5.2	(4.8)	G > B**
Attention problems	7.6	(4.6)	B > G*		17.4	(12.0)	B > G***		6.4	(3.8)	
Rule-breaking behaviour	4.0	(4.0)	B > G*		3.2	(4.1)	B > G*	O > Y*	5.5	(4.6)	
Aggressive behaviour	9.7	(7.3)	B > G*		8.2	(8.8)	B > G***		8.5	(5.8)	
Internalizing problems	13.3	(9.0)	G > B*		9.2	(7.5)		O > Y**	15.5	(12.2)	G > B***
Externalizing problems	13.7	(10.4)	B > G*		11.5	(11.8)	B > G***		13.9	(9.5)	
Total problems	46.7	(24.3)			44.0	(27.9)	B > G***		49.9	(30.7)	G > B*

Mean (SD) and the effects of sex and age on the ASEBA scales.

^1 ^Effects were analysed using the general linear model (GLM) in SPSS, and the significant effects are shown. There were no significant interaction effects (sex × age).

*P < 0.05 and false-discovery rate (FDR) < 0.14, **P < 0.01 and FDR < 0.05, ***P < 0.001 and FDR < 0.002, G = girls, B = boys, Y = younger (6-10 years), O = older (11-17 years).

### Data analysis

Statistical analyses (except calculation of false discovery rate) were conducted using SPSS 15.0 for Windows. The effects of sex, age group (6-10 or 11-17 years), and sex × age group on all the HoNOSCA and ASEBA scales were analysed using the general linear model (GLM) in SPSS. The associations between the HoNOSCA scales and the ASEBA scales were investigated using Pearson correlation analyses. In some cases, where the distributions of both variables were extremely skewed, the significant results were also analysed with non-parametric correlations (Kendall's tau). Since we performed a large number significance tests, we also calculated false discovery rates (FDR) for each table. Instead of looking at the probability of at least one type I error as in Bonferroni's correction, FDR controls the expected proportion of type I errors among all responses reported as significant [31]. To handle non-structured dependence among the variables, a variant of FDR [32] that is based on rotation testing [33] were utilized. This approach is based on regression modelling with multiple responses and a rotation testing analysis was therefore performed for each column in Tables 2, 3, 4 and 5. The rows in these tables correspond to the response variables in the regression model. For each table, we found the FDR limits that correspond to the ordinary significance levels so that all the analyses were covered. The FDR calculations were conducted using a Matlab program [34]. Regression analyses were conducted to determine how the variability in HoNOSCA (dependent variable) could be explained by scores on the three ASEBA forms: CBCL, TRF, and YSR (independent variables). The change in the explained variance, caused by adding the ASEBA variables, is denoted "ΔR^2 ^ASEBA". "ΔR^2 ^alone" is the result of adding only a single ASEBA variable. The unique variance "ΔR^2 ^unique" was obtained by adding a single ASEBA variable to a model that also contained the other ASEBA variable(s). The collinearity between the independent variables was investigated and was not considered a problem because all intercorrelations were less than 0.63. There was no significant interaction with age group (6-10 years or 11-17 years) on the association between the independent and dependent variables in the regression analyses for any of the models. Therefore, we analysed the models with the CBCL and TRF (not the YSR) for the whole group, with ages spanning 6-17 years.

**Table 4:** Correlations with ASEBA broad-band scales.

	Internalizing			Externalizing			Total problems		
HoNOSCA scales:	CBCL	TRF	YSR	CBCL	TRF	YSR	CBCL	TRF	YSR
***n***	153	153	75	153	153	75	153	153	75
1. Aggressive behaviour	0.10	-0.10	0.10	0.62***	0.46***	0.46***	0.46***	0.34***	0.27*
2. Overactivity or attention problems	-0.09	-0.16*	0.001	0.41***	0.39***	0.36**	0.35***	0.41***	0.21
3. Self-injury	0.17*	0.10	0.63***	0.07	-0.04	0.44***	0.06	-0.09	0.58***
4. Drug or alcohol misuse	-0.07	-0.07	0.14	0.19*	0.18*	0.43***	0.02	0.04	0.24*
5. Scholastic problems	-0.02	0.04	-0.13	0.25**	0.22**	0.09	0.25**	0.37***	0.01
6. Somatic problems	0.10	0.12	-0.15	-0.04	-0.05	-0.07	0.10	0.07	-0.14
7. Abnormal thoughts or perceptions	0.11	-0.11	0.34**	-0.08	-0.13	0.12	-0.04	-0.18 *	0.31**
8. Psychosomatic symptoms	0.19*	0.11	-0.05	-0.17*	-0.18*	-0.34**	-0.01	-0.14	-0.22
9. Emotional symptoms	0.43***	0.28***	0.52***	-0.14	-0.19*	0.10	0.06	-0.16	0.33**
10. Peer problems	0.32***	0.26**	0.20	0.18*	0.17*	0.04	0.37***	0.32***	0.13
11. Self-care problems	0.01	-0.03	-0.28*	0.02	-0.04	-0.15	0.14	0.06	-0.25*
12. Family problems	0.004	-0.05	0.06	0.20*	0.17*	0.21	0.09	0.08	0.09
13. Poor school attendance	0.23**	0.19*	0.29*	0.24**	0.15	0.35**	0.21*	0.13	0.29*
Total Score (sum scale 1-13)	0.33***	0.13	0.35**	0.41***	0.27**	0.44***	0.49***	0.32***	0.41***

Pearson correlations between the HoNOSCA scales and ASEBA internalizing, externalizing, and total problems scales.

*P < 0.05 and false-discovery rate (FDR) < 0.15, **P < 0.01 and FDR < 0.03, ***P < 0.001 and FDR < 0.006.

Bold numbers are correlations expected to be high (HoNOSCA scale 1 *vs *ASEBA externalizing; HoNOSCA scale 9 *vs *ASEBA internalizing; HoNOSCA total score *vs *ASEBA total problems).

**Table 5:** Correlations with ASEBA syndrome scales.

	**III. Somatic problems**^**1**^			IV. Social problems			VI. Attention problems		
HoNOSCA scales:	CBCL	TRF	YSR	CBCL	TRF	YSR	CBCL	TRF	YSR
***n***	153	153	75	153	153	75	153	153	75
1. Aggressive behaviour	0.02	-0.11	0.13	0.31***	0.17*	0.05	0.35***	0.33***	0.26*
2. Overactivity or attention problems	-0.07	-0.13	0.03	0.34***	0.19*	0.04	0.61***	0.58***	0.45***
3. Self-injury	0.13	0.08	0.50***	-0.05	-0.09	0.25*	-0.12	-0.22**	0.32**
4. Drug or alcohol misuse	-0.07	-0.04	0.09	-0.13	-0.10	0.02	-0.09	-0.02	0.004
5. Scholastic problems	-0.12	-0.08	-0.19	0.25**	0.19*	0.09	0.49***	0.51***	0.28*
6. Somatic problems	-0.03	-0.02	-0.12	0.25**	0.19*	-0.12	0.15	0.06	-0.15
7. Abnormal thoughts or perceptions	0.07	0.06	0.21	-0.09	-0.15	0.26*	-0.13	-0.17*	0.23*
8. Psychosomatic symptoms	0.25**	0.21**	**0.12**	0.03	-0.04	-0.18	-0.10	-0.20*	-0.27*
9. Emotional symptoms	0.28***	0.12	0.35**	0.03	-0.02	0.22	-0.26**	-0.37***	0.07
10. Peer problems	0.09	-0.01	0.09	0.59***	0.52***	0.24*	0.26**	0.19*	0.06
11. Self care problems	-0.05	-0.08	-0.28*	0.24**	0.13	-0.17	0.26**	0.14	-0.15
12. Family problems	-0.06	-0.04	-0.07	0.05	0.04	-0.02	0.03	0.04	-0.01
13. Poor school attendance	0.18*	0.23**	0.37**	0.04	0.06	0.09	0.04	0.002	0.11
Total Score (sum scale 1-13)	0.14	0.02	0.26*	0.47***	0.29***	0.18	0.41***	0.27**	0.30**

Pearson correlations between the HoNOSCA scales and selected ASEBA syndrome scales (attention problems, somatic problems, and social problems).

*P < 0.05 and false-discovery rate (FDR) < 0.18, **P < 0.01 and FDR < 0.06, ***P < 0.001 and FDR < 0.004.

^1 ^The syndrome scale "somatic problems" is part of "internalizing problems" in Table 4.

Bold numbers are correlations expected to be high (HoNOSCA scale 2 *vs *ASEBA attention problems; HoNOSCA scale 8 *vs *ASEBA somatic problems; HoNOSCA scale 10 *vs *ASEBA social problems).

### Ethics

The data collection was based on the informed written consent of the participants. The study was approved by the Regional Committees for Medical Research Ethics, Southern and Northern Norway, and the Norwegian Data Inspectorate.

## Results

### Correlations between HoNOSCA and ASEBA scores

The inter-informant correlations between the ASEBA *total problems *were: CBCL and TRF = 0.30 (*P *< 0.001), CBCL and YSR = 0.50 (*P *< 0.001), and TRF and YSR = 0.14 (*P *= 0.222). The correlations between scores on the HoNOSCA scales and scores on the broadband ASEBA scales are presented in Table 4. The correlations between the HoNOSCA *total score *and the ASEBA (CBCL, YSR, and TRF) *total problems *were all medium. HoNOSCA scale 1 *aggressive behaviour *had large or medium positive correlations with the ASEBA *externalizing problems*, and no significant correlation with the ASEBA *internalizing problems*. HoNOSCA scale 9 *emotional symptoms *had large, medium or small positive correlations with the ASEBA *internalizing problems*, and no significant positive correlation with the ASEBA *externalizing problems*.

The correlations between HoNOSCA scale 2 *overactivity or attention problems*, scale 8 *psychosomatic symptoms*, and scale 10 *peer problems *and the selected ASEBA syndrome scales *attention problems*, *somatic problems*, and *social problems*, respectively, are presented in Table 5. HoNOSCA scale 8 *psychosomatic symptoms *had low correlations with the CBCL and TRF *somatic problems*, and did not correlate significantly with the YSR *somatic problems*. Table 6 shows how HoNOSCA scale 3 *self-injury*, HoNOSCA scale 4 *drug and alcohol misuse*, HoNOSCA scale 7 *abnormal thoughts or perceptions*, and HoNOSCA scale 13 *poor school attendance *correlated with the sum of the relevant ASEBA items in the oldest age group.

**Table 6:** Correlations with ASEBA items.

HoNOSCA scales:	CBCL *n *= 92	TRF *n *= 92	YSR *n *= 75
	***r***	***r***	***r***
3. Self-injury *vs *ASEBA items 18 + 91	0.37***	0.36***	0.63***
4. Drug or alcohol misuse *vs *ASEBA items 2 (CBCL/YSR) + 105	0.61***	0.26*	0.43***
7. Abnormal thoughts or perceptions *vs *ASEBA items 9 + 34 + 40 + 70 + 85 + 89	0.21*	0.03	0.48***
13. Poor school attendance *vs *ASEBA items 98 (TRF) + 101	0.57***	0.45***	0.54***

Pearson correlation between some HoNOSCA scales and sum of similar items in ASEBA (age group: 11-17 years).

*P < 0.05, ***P < 0.001.

Analysis is of the oldest age group because these HoNOSCA scales were rated zero for 90%-100% of the children in the 6-10 year age group.

All correlations in this table were also computed with Kendall's tau in SPSS software, giving no higher P values, except for HoNOSCA scale 13 poor school attendance vs TRF (*P *= 0.001).

Two methodological issues were specifically studied: whether the time difference between the ratings by the ASEBA informants and the clinician were related to the HoNOSCA results and whether the availability of the ASEBA results to the clinician were related to the HoNOSCA results. No significant main or interaction effects were found for the time difference or availability in relation to the HoNOSCA *total score*.

### Prediction of HoNOSCA scores by the ASEBA informants' scores

Table 7 shows how the scores given by the different ASEBA informants predicted the clinician-rated HoNOSCA scores. Sex and age were corrected for in the first block (included in the total explained variance, R^2^, in Table 7). The CBCL and TRF *total problems *together (ΔR^2 ^ASEBA) explained 27% of the variance in the HoNOSCA *total score*. The unique explained variance (ΔR^2 ^unique) was 16% for the CBCL (when the TRF was already included in the model) and 4% for the TRF (when the CBCL was already included). The CBCL alone (ΔR^2 ^alone) explained 23%, and the TRF alone explained 11% of the variance in the HoNOSCA total score. For the oldest group (11-17 years), all three ASEBA measures (CBCL, TRF, and YSR) together explained 23% of the variance in the HoNOSCA *total score*. However, only the CBCL *total problems *had any unique explained variance (ΔR^2 ^unique = 0.06).

**Table 7:** Regression analyses

Dependent variables	Independent variables	Age group	Form	ΔR2 alone (if first)	ΔR2 unique (if last)	ΔR2 ASEBA	R2 Total
HoNOSCA total score	Total problems	6-17 years	CBCL	0.23***	0.16***	0.27***	0.31***
			TRF	0.11***	0.04**		
		11-17 years	CBCL	0.19***	0.06*	0.23***	0.28***
			TRF	0.07*	0.01		
			YSR	0.14**	0.03		
HoNOSCA scale 1 *aggressive behaviour*	Externalizing problems	6-17 years	CBCL	0.35***	0.22***	0.38***	0.44***
			TRF	0.16***	0.03*		
		11-17 years	CBCL	0.37***	0.14***	0.38***	0.41***
			TRF	0.16***	< 0.01		
			YSR	0.20***	< 0.01		
HoNOSCA scale 2 *Overactivity or attention problems*	Attention problems	6-17 years	CBCL	0.32***	0.13***	0.42***	0.48***
			TRF	0.28***	0.09***		
		11-17 years	CBCL	0.29***	0.09**	0.41***	0.41***
			TRF	0.24***	0.07**		
			YSR	0.21***	0.01		
HoNOSCA scale 8 *psychosomatic symptoms*	Somatic problems	6-17 years	CBCL	0.05**	0.03*	0.08**	0.10**
			TRF	0.05**	0.02		
		11-17 years	CBCL	< 0.01	< 0.01^1^	0.04	0.14
			TRF	0.03	0.03		
			YSR	< 0.01	< 0.01		
HoNOSCA scale 9 *emotional symptoms*	Internalizing problems	6-17 years	CBCL	0.13***	0.09***	0.15***	0.25***
			TRF	0.06**	0.01		
		11-17 years	CBCL	0.09**	< 0.01	0.22***	0.32***
			TRF	0.09**	0.03		
			YSR	0.18***	0.09**		
HoNOSCA scale 10 *peer problems*	Social problems	6-17 years	CBCL	0.35***	0.13***	0.41***	0.42***
			TRF	0.28***	0.06***		
		11-17 years	CBCL	0.23***	0.04*	0.35***	0.37***
			TRF	0.30***	0.12**		
			YSR	0.06*	< 0.01		

Results from several multiple linear regression analyses explaining the variance in selected HoNOSCA scales from similar ASEBA scales completed by parents (CBCL), teachers (TRF), and patients 11-17 years (YSR), controlled for sex and age (continuous variable).

*P < 0.05, **P < 0.01, ***P < 0.001.

^1 ^β is negative for CBCL in this model (age group 11-17 years); in all the other regression models, the ASEBA predictors had positive β values.

The ASEBA scores explained a large proportion of the variance in HoNOSCA scale 1 *aggressive behaviour *(ΔR^2 ^ASEBA = 0.38) and HoNOSCA scale 2 *overactivity or attention problems *(ΔR^2 ^ASEBA = 0.41) in the models both with and without the YSR. The unique prediction of the parents was higher in these models than that of the teachers or young people. The ASEBA scores also explained a large proportion of the variance in HoNOSCA scale 10 *peer problems*. For the oldest age group, the TRF *social problems *had the highest unique prediction. The ASEBA scores explained somewhat less of the variance in HoNOSCA scale 9 *emotional symptoms*, and YSR had the highest unique prediction for the oldest age group. The ASEBA did not predict the clinicians' ratings of HoNOSCA scale 8 *psychosomatic symptoms *for the oldest age group, and CBCL and TRF explained 8% of the variance in the total (all ages) group.

## Discussion

In this study, we compared the total score and nine of the 13 scales of the clinician-rated HoNOSCA in routine clinical use with relevant scales or combinations of items in the ASEBA. The general finding was that mental health rated by clinicians using the HoNOSCA correlated, as expected, with the mental health rated by parents, teachers, and young people themselves using the ASEBA. These results support the validity of the HoNOSCA. The mean HoNOSCA *total score *of 12.0 (SD 4.6) in our study was similar to the results of other CAMHS outpatient studies [7,12,18,35], indicating that the sample was comparable to other outpatient samples. We found skewed distributions towards low mean scores on most of the 13 HoNOSCA scales. This most probably indicates that children and adolescents attending outpatient CAMHS have severe problems on some, but far from all of the HoNOSCA scales. Skewed results, with low scores on a scale, may imply reduced sensitivity to change and low ability to measure outcome with these single scales. However, the single scales are rarely used to measure outcome. The HoNOSCA *total score *may be more appropriate to measure change, also across different diagnostic groups [7,12]. The ASEBA *total problems *and syndrome scale scores in our sample were clearly higher than the scores reported for a general population sample in Norway [36,37] and consistent with Scandinavian results from an outpatient clinical sample [38] but slightly lower than those for a clinical sample reported in the ASEBA manual (Appendix D) [26].

### Concurrent validity of the HoNOSCA

Our finding that the HoNOSCA *total score *had medium correlation (*r *= 0.49) with the CBCL *total problems *reflects the correlations reported by others with the SDQ *total difficulties score *assigned by parents [18,24] and with the CBCL [25]. Our results show higher correlations than the results of a meta-analysis [27] (including both clinical and non-clinical samples), with a mean correlation of 0.28 between the scores of parents and those of mental health workers. A correlation of 0.41 between the HoNOSCA *total score *and the YSR *total problems *and a correlation of 0.32 between the HoNOSCA *total score *and the TRF *total problems *are similar to the correlations reported in studies that compared the HoNOSCA and SDQ, with ratings by young people and teachers [24], and in a study that compared the HoNOSCA and TRF [25]. They are also similar to the mean correlations previously found between the scores of mental health workers and self-reports (0.27), and between the scores of mental health workers and those of teachers (0.34) [27]. In general, greater agreement has been found when reporting under-controlled (externalizing) problems (mean *r *= 0.41) than when reporting over-controlled (internalizing) problems (mean *r *= 0.32) [27], and our findings are similar. The results for the more specific scales showed correspondence between the HoNOSCA and ASEBA on similar phenomena with medium-large correlations across the different informants, and small negative or no correlations on divergent phenomena. An exception was HoNOSCA scale 8 *psychosomatic symptoms*, which produced only small correlation coefficients when compared with *somatic problems *in the CBCL and TRF, and no significant correlation with *somatic problems *in the YSR.

Brann [25] compared HoNOSCA scale 1 *aggressive behaviour *and the *externalizing problems *of the CBCL with a correlation of *r *= 0.46 (we found *r *= 0.62) and TRF with a correlation of *r *= 0.57 (we found *r *= 0.46). He further compared HoNOSCA scale 9 *emotional symptoms *and the *internalizing problems *of the CBCL with a correlation of *r *= 0.33 (we found *r *= 0.43) but found no significant correlation with the TRF, contrary to our finding (*r *= 0.28).

The clinicians' rating of HoNOSCA scale 3 *self-injury *had a large correlation with similar items in the YSR, and a medium correlation with those in the CBCL and TRF. This is consistent with the finding that deliberate self-harm is often a hidden problem in adolescents, of which parents and teachers are unaware [39,40]. The clinicians' rating of HoNOSCA scale 7 *abnormal thoughts or perceptions *had a medium correlation with the items from the YSR, a small correlation with the CBCL and had no correlation at all with the TRF. The selected ASEBA items may not compare well with the clinicians' terms "hallucinations/abnormal perceptions" and "delusions/abnormal thoughts". However, the medium correlation with the YSR is interesting. Although we have found a substantial correspondence between the HoNOSCA scales and the similar ASEBA scales, the results cannot be said to overlap. This underlines the importance of a multi-informant assessment strategy.

### Prediction of HoNOSCA scores by ASEBA informants' scores

When they are the only informants, parents are good predictors of the HoNOSCA *total score *and the three scales: scale 1 *aggressive behaviour*, scale 2 *overactivity or attention problems*, and scale 10 *peer problems*. However, teachers added considerably to the prediction of HoNOSCA scale 2 *overactivity or attention problems *and scale 10 *peer problems*. For the oldest age group, teachers were even better than parents in predicting the peer problems scored by the clinician. Young people best predicted HoNOSCA scale 9 *emotional symptoms*, whereas parents and teachers did not add any more to the young people's information. Without the young people's information, parents were better than teachers in predicting the clinicians' rating of emotional symptoms. For the five HoNOSCA scales with similar ASEBA scales, the CBCL provided unique predictions of all the HoNOSCA scales, the TRF provided unique predictions of three of the HoNOSCA scales, and the YSR provided a unique prediction of one HoNOSCA scale. It is noteworthy that all the informants--parents, young people, and teachers--provided at least some unique information for predicting the HoNOSCA scores.

### Methodological issues

This was a naturalistic study of the HoNOSCA and ASEBA scales in ordinary outpatient CAMHS clinics, with the advantage of analysing real patients, clinicians, and clinics. However, the procedures had to be adapted to the clinical setting, and it was difficult to obtain full data sets at the right times. Considerable variation was found between the clinics in patient participation, the number of participating clinicians, and the number of patients rated by each clinician. The ASEBA forms were collected as part of our research protocol, and we did not intend ASEBA to be used for clinical purposes. It is a weakness of the study that we do not know whether some clinicians used the information from the ASEBA when they rated the HoNOSCA. The availability of the ASEBA results to the clinicians had no apparent effect. This indicates that they generally did not use the ASEBA information. The clinics trained the clinicians to use the HoNOSCA, and 75% of the patients were rated by a trained clinician. That some clinicians lacked training may have biased the results, but we have no information from reliability tests about how training influences the inter-rater reliability of the HoNOSCA. Seventy-two per cent of the patients were rated by a clinician who had participated in a larger study of the inter-rater reliability of the HoNOSCA, in which its reliability was found to be quite satisfactory [16]. Those who did not participate in the reliability study were clinicians working at two CAMHS clinics that were recruited after the reliability study or were at leave at the time of the reliability study. Our focus was on the assessment methods, and an essential topic in relation to generalizability of our results is the severity of the patient symptoms and the variability in the sample. In our study sample, the HoNOSCA *total score *was close to those reported in other studies of outpatient samples [7,12,14,18,35]. However, the low scores and restricted range on most single scales is a limitation for our correlation analyses where we use single scales. We studied the HoNOSCA as an assessment method, not as an outcome measure. Other studies have evaluated the HoNOSCA as an outcome measure [7,12,14,18,22,35,41] or used it in treatment studies [42-45], and have found it to be sensitive to change. One of the strengths of our study is that we could compare the clinicians' ratings (HoNOSCA) with data from several other informants--in this case parents, teachers, and the young people themselves.

### Clinical implications

In ordinary outpatient CAMHS, the HoNOSCA is a broad measure that is well suited to assessing the severity and type of mental health symptoms, problems, and impairment in children and adolescents aged 6-17 years. A multi-informant strategy, which includes clinicians as well as parents, teachers, and adolescents, is recommended. More-specific measures should be included as appropriate.

## Conclusions

The HoNOSCA *total score *and eight of the nine HoNOSCA scales investigated were found to have good concurrent validity compared with the ratings by parents (CBCL), teachers (TRF), and young people (YSR), in a clinical sample. All these informants contributed unique information in relation to the clinician-rated HoNOSCA, indicating that the HoNOSCA ratings reflect unique perspectives from multiple informants.

## Competing interests

The authors declare that they have no competing interests.

## Authors' contributions

KHB, SH, and SK initiated the study. KHB and SK collected the data. KHB and SH analysed the results and drafted the paper. ØL (statistician) analysed the results and drafted the text on the statistical analyses. All authors commented on the drafts of the paper, and read and approved the final manuscript.
